# Analyses of Long Noncoding RNA and mRNA Profiles in Subjects with the Phlegm-Dampness Constitution

**DOI:** 10.1155/2021/4896282

**Published:** 2021-12-10

**Authors:** Lidan Dong, Yanfei Zheng, Dan Liu, Fuhong He, Kaiki Lee, Lingru Li, Qi Wang

**Affiliations:** ^1^National Institute of Traditional Chinese Medicine Constitution and Preventive Medicine, Beijing University of Chinese Medicine, Beijing 100029, China; ^2^Key Laboratory of Genomics and Precision Medicine, Collaborative Innovation Center of Genetics and Development, Beijing Institute of Genomics, Chinese Academy of Sciences, Beijing 100101, China

## Abstract

**Background:**

Constitution in traditional Chinese medicine (TCM) plays a key role in the genesis, development, and prognosis of diseases. Phlegm-dampness constitution (PDC) is one of the nine constitutions in TCM, susceptible to metabolic disorders, which is mainly manifested by profuse phlegm, loose abdomen, and greasy face. Epidemiologic, genomic, and epigenetic studies have been carried out in previous works, confirming that PDC represents a distinctive population with microcosmic changes related to metabolic disorders. However, whether long noncoding RNAs (lncRNAs) play a regulatory role in metabolic disease in subjects with PDC remains largely unknown. We aimed to investigate distinct lncRNA and mRNA expression signatures and lncRNA-mRNA regulatory networks in the phlegm-dampness constitution (PDC).

**Methods:**

The peripheral blood mononuclear cells (PBMCs) were isolated from the subjects with PDC (*n* = 13) and balanced constitution (BC) (*n* = 9). The profiles of lncRNAs and mRNAs in PBMCs were analyzed using microarray and further validated with RT-qPCR. Subsequently, pathway analysis was performed to investigate the function of differentially expressed mRNAs by using Ingenuity Pathway Analysis (IPA).

**Results:**

Results suggested that some mRNAs, which were regulated by the differentially expressed lncRNAs, were mainly enriched in lipid metabolism and immune inflammation-related pathways. This was consistent with the molecular characteristics of previous studies, indicating that the clinical characteristics of metabolic disorders in PDC might be regulated by lncRNAs. Furthermore, by making coexpression network construction as well as *cis*-regulated target gene analysis, several lncRNA-mRNA pairs with potential regulatory relationships were identified by bioinformatic analyses, including RP11-317J10.2-CA3, RP11-809C18.3-PIP4K2A, LINC0069-RFTN1, TTTY15-ARHGEF9, and AC135048.13-ORAI3.

**Conclusions:**

This study first revealed that the expression characteristics of lncRNAs/mRNAs may be potential biomarkers, indicating that the distinctive physical and clinical characteristics of PDC might be partially attributed to the specific expression signatures of lncRNAs/mRNAs.

## 1. Background

Constitution in traditional Chinese medicine (TCM) plays an important role in the initiation, development, and prognosis of diseases. The term “constitution” is coined to indicate the distinct TCM entity or “type.” Based on clinical presentations, healthy people can be classified into nine constitutions according to the concept of TCM, including one balanced constitution (BC) and eight biased constitutions [1–3]. Each constitution exhibits specific physical features and psychological characteristics, and each biased constitution demonstrates a specific predisposition to certain diseases and should be treated differently. Phlegm-dampness constitution (PDC), which is one of the biased constitutions, is a group of individuals who tend to develop diseases, or those in a dysfunctional/subhealth status but do not reach the diagnostic criteria for a specified disorder, and these subjects with PDC are usually obese and not considered healthy in modern medicine [4, 5]. Moreover, through years of clinical practice, we summarized the common characteristics of the PDC, including obesity, abundant sputum, greasy and soft lower abdomen, oily skin in the face, chest distress, sticky and sweet taste in the mouth, and slippery pulse [6]. Accumulating evidence has suggested that PDC plays an important role in the prevention stage of multiple metabolic diseases [7, 8]. Therefore, it is necessary to identify which population tends to have the potential risk of metabolic disorders and what molecular markers can be used to identify subjects with PDC.

The epidemiological investigation, single-nucleotide polymorphisms (SNPs), genomics, and methylation investigation have previously confirmed that subjects with PDC belong to a population with clinical features of microcosmic changes related to metabolic disorders [8–10]. A previous study demonstrated that certain differentially expressed genes in the peripheral blood mononuclear cells (PBMCs) of individuals with PDC were associated with the adipocytokine signaling pathway, insulin signaling pathway, and fatty acid elongation in mitochondria [11–13]. In a separate report, methylation in the SQSTM1, DLGAP2, DAB1, HOXC4, and SMPD3 genes were shown to be associated with specific constitution types (diabetes mellitus, obesity, and so on), and differentially methylated genes are abundantly enriched in multiple metabolic pathways [14, 15]. In addition, individuals with PDC, who have specific gene expression signatures (such as SOCS3, ACSL4, CLU, and ABCG1), are more susceptible to metabolic disorders [16].

These previous reports have provided insights into the molecular basis of individuals with PDC; however, the roles of long noncoding RNAs (lncRNAs) in metabolic disorders in the PDC remain poorly understood. lncRNAs are a large group of noncoding transcripts that are more than 200 nucleotides in length [17]. lncRNAs have been extensively reported to be involved in gene regulation and cellular processes through a diversity of mechanisms, such as epigenetic, transcriptional, and posttranscriptional levels [18–20]. Numerous studies have indicated that dysregulation of lncRNAs is highly associated with a wide variety of diseases, such as heart failure, myocardial infarction (MI), cardiovascular disease, and diabetes [21–23]. For example, lncRNAs Gadd7 is necessary for lipid- and general oxidative stress-mediated cell death and its depletion or suppression may be a promising approach to treat or prevent heart failure [24]. A previous study has demonstrated that a small SNP mutation (rs2301523) in MIAT can upregulate the expression of MIAT, leading to a high risk of MI [25]. Besides, Shi and coworkers have reported that lncRNA GAS5 is a target for therapeutic intervention in the management of type 2 diabetes [26]. These previous reports have confirmed that lncRNAs play a crucial role in metabolic disorders. However, the expression patterns, potential targets, and functions of lncRNAs in the development and pathogenesis of the metabolic disorders in subjects with PDC remain largely unexplored.

In the present study, we analyzed the expression profiles of lncRNAs and mRNAs in PBMCs. The key lncRNAs and target genes have the molecular characteristics of metabolic disorder tendency in the PDC, which were verified by RT-qPCR in independent samples. Besides, bioinformatic analyses were performed to reveal the regulatory role of lncRNAs in metabolic disorders of subjects with PDC. Collectively, our study first pointed out which populations with PDC were susceptible to metabolic disease from the perspective of lncRNA.

## 2. Materials and Methods

### 2.1. Study Subjects

Subjects in this study were recruited from the Physical Examination Center, Hong Yi Tang of Traditional Chinese Medicine, in March 2014. This study was performed in compliance with the Declaration of Helsinki, and the research protocol was approved by the ethics committee of the Beijing University of Traditional Chinese Medicine (2012BZHYLL0301). Written informed consent was obtained from all participants. Individuals with BC (*n* = 9) and PDC (*n* = 13) were chosen using a standardized questionnaire (Wang Qi's Body Constitution Classification Questionnaire-Chinese version, frequent code: ZYYXH/T 157-2009) (Table 1) issued by the Chinese Association of Traditional Chinese Medicine and confirmed by Chinese medicine clinical practitioners (*n* = 15). Any differences in the opinions of these practitioners were resolved after careful discussion. The criteria for inclusion and exclusion of patients are as follows:

1. The inclusion criteria include the following: (1) natural population aged 18-65 in Beijing; (2) according to the “TCM Constitution Classification and Judgment Standard” approved by the Chinese Academy of Traditional Chinese Medicine, those who meet the judgment criteria of all constitutions and peaceful constitutions; (3) signed informed consent; (4) have no medical diseases, and (5) not taking medication.
2. The exclusion criteria include the following: (1) failure to meet diagnostic criteria or inclusion criteria; (2) people with mixed physique; (3) those who have been diagnosed with a chronic disease; and (4) pregnant women.

### 2.2. RNA Extraction

Peripheral blood (6 mL) was collected by phlebotomy and stored in sodium citrate vacuum tubes. The isolation of PBMCs was performed within 4 h after the blood collection by density-gradient centrifugation using Lymphoprep (Sigma, Life Science, USA). The isolated PBMCs were treated with TRIzol reagent (Invitrogen, Carlsbad, CA, USA) to avoid RNA degradation and then stored at -80°C. Total RNA was extracted and purified from each sample in blood in 2014, according to the manufacturer's instructions. The RNA concentration was determined using a NanoDrop 2000 spectrophotometer (Thermo Fisher Scientific, USA), and the RNA integrity was analyzed by agarose gel electrophoresis.

### 2.3. Microarray Analysis

This experiment was performed technically as a dual channel. Total RNA was hybridized to lncRNA+mRNA Human Gene Expression Microarray V4.0 (4∗180 K) (CapitalBio). An improved Eberwine's linear RNA amplification approach and enzymatic reaction mentioned in a CapitalBio cRNA Amplification and Labeling Kit from CapitalBio were utilized to get greater yields of cDNA labeled with a fluorescent dye (Cy3-dCTP and Cy5-dCTP) [27]. Feature Extraction software (version10.7.1.1) from Agilent Technologies was adopted to produce raw data from array images. The quality control, normalization, and summarization of mRNA and lncRNA data were carried out by using GeneSpring software (v13.0) from Agilent. The differentially expressed lncRNAs (DElncRNAs) and mRNAs (DEmRNAs) were identified based on the fold change and the “limma” package in R software. The mRNAs and lncRNAs with a *P* < 0.05 and ∣fold change | ≥1.5 were considered differentially expressed in populations with PDC and BC.

### 2.4. Coexpression Network of lncRNAs with mRNAs and Associated Functional Prediction

To explore the potential functions of lncRNAs, a coexpression network was constructed between the expression values of mRNAs and lncRNAs based on the correlation analysis. An absolute value of Pearson's correlation coefficient (|PCC | >0.7 and a *P* < 0.05) was considered statistically significant. A strong correlation (∣PCC | >0.7) is a commonly selected threshold in research [28, 29]. Subsequently, the enriched functions of mRNAs could be used to predict the functions of lncRNAs that were coexpressed with these mRNAs. Datasets of target genes of lncRNAs were analyzed as input data to identify enriched signaling pathways and biological functions using Ingenuity Pathway Analysis (IPA) (Ingenuity System Inc., USA). The threshold of significance of pathways was defined by the *P* value, and the selection criterion for significant IPA pathway terms was *P* < 0.05. The enriched functional terms were used as the predicted functional terms of given lncRNAs. The interactions between lncRNAs and mRNAs were analyzed, and the coding-noncoding gene coexpression network (CNC) was constructed with Cytoscape (version 3.7.0) software.

### 2.5. Prediction of lncRNA Target Genes

Target genes of lncRNAs were predicted including two aspects as follows: (a) the CNC (∣PCC | >0.7) of DElncRNAs and DEmRNAs was constructed; (b) coding genes, 300 kb upstream and downstream of DElncRNAs, were used to predict the functions of lncRNAs. We choose 300 kb upstream and downstream of lncRNAs as criteria that are a commonly selected threshold in research (see references as follows for details [30, 31]). The intersection of the DElncRNA-DEmRNA pairs was obtained, and these DEmRNAs were the target genes of DElncRNAs. The expression levels of the DElncRNAs and coding genes were used to analyze their co-expression relationships. ∣PCC | >0.7 and *P* < 0.05 were considered to be correlated expression. We applied Cytoscape (version 3.7.2) to visualize the networks. The degree was performed using the CentiScaPe in Cytoscape to illuminate the most important nodes in the network [32].

### 2.6. Validation by RT-qPCR of Key lncRNAs and mRNAs in Independent Samples

According to the inclusion and exclusion criteria, nine subjects with PDC and 12 BC were incorporated in our study in June 2019. Clinical information of these 21 subjects is presented in Table S3. All patients provided informed consent to participate in this study. Quantification was performed with a reaction process consisting of two steps, including reverse transcription (RT) and RT-qPCR, to confirm the microarray results. RT-qPCR for lncRNAs and mRNAs was performed as previously described [33]. Primers used for RT-qPCR are listed in Table S1. Moreover, *β*-actin was selected as the housekeeping gene. The relative expressions of target genes were calculated using the 2^-*ΔΔ*Ct^ method [34].

### 2.7. Statistical Analysis

The numerical data were expressed as the mean ± standard deviation (SD). Statistical comparisons between groups of normalized data were performed using the “limma” package of R software, *t*-test, or Mann–Whitney *U* test when appropriate. Statistical comparisons between paired PDC and BC were performed using a paired *t*-test. A *P* < 0.05 was considered statistically significant with a 95% confidence level. Statistical analyses were performed using SPSS software 25.0, and mappings were performed using the R 3.3.1 software (the R Foundation of Statistical Computing).

## 3. Results

### 3.1. Differentially Expressed lncRNAs and mRNAs in the PDC

To determine the transcriptome characteristics and which lncRNAs could be used to PDC from those with BC, we surveyed the lncRNA and mRNA expression profiles of subjects with a total of 22 samples from PDC (*n* = 13) and BC (*n* = 9) using microarray analysis (NCBI GEO accession number GSE158042). The purity and integrity of Total RNA were good, and these samples were used for the microarray analysis in 2014. The distributions of lncRNAs and mRNAs are shown in Fig. S1. All subjects were Chinese Han individuals, 20-53 years old, including both genders. There was no significant difference in age (*P* = 0.78), sex (*P* = 0.94), and body mass index (BMI) (*P* = 0.12) between the two groups of subjects.  Table 2 summarizes the main clinical characteristics of enrolled patients with no medical records of diagnosed diseases.

**Figure 1:**
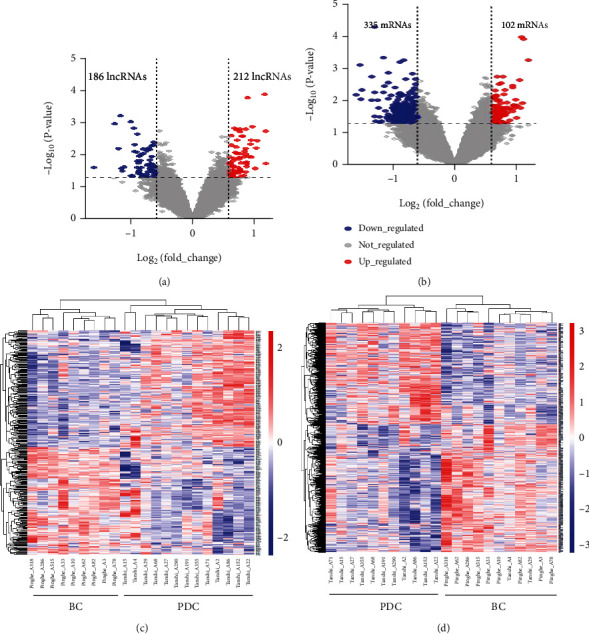
The hierarchical clustering heatmaps and volcano plots of differentially expressed lncRNAs and mRNAs. The row and column represent differentially expressed lncRNAs (a and c)/mRNAs (b and d) and samples, respectively, in the heatmaps (*P* < 0.05, ∣fold change | ≥1.5) in phlegm-dampness constitution (PDC) compared with balanced constitution (BC). Red and blue indicate up- and downregulation, respectively. -3, -1, 0, 1, and 3 represent fold changes in the corresponding spectrum.

After the microarray analysis, the raw total count of lncRNA and mRNA after normalization is 22,621 and 31,860, respectively. A total of 398 differentially expressed lncRNAs and 437 differentially expressed mRNAs were identified based on |fold change | ≥1.5 and *P* < 0.05 in the PDC compared with the BC. The volcano plot, respectively, is shown in Figure 1(a) and 1(b). The sample grouping was largely consistent when using DElncRNAs (Figure 1(c)) or DEmRNAs (Figure 1(d)). Among them, it is known that TMPO is associated with the progression of type 1 diabetes [35]. ACSL3 plays a key role in lipid biosynthesis and fatty acid degradation [36]. Among the dysregulated lncRNAs, suppression of TTTY15 attenuates hypoxia-induced cardiomyocyte injury by targeting miR-455-5p [37]. The above-mentioned genes are related to metabolic disorders, which are consistent with previous studies, [10, 12, 14], and they were significantly expressed in subjects with PDC in this study.

### 3.2. Functional Analysis of Differentially Expressed mRNAs in the PDC

**Figure 2:**
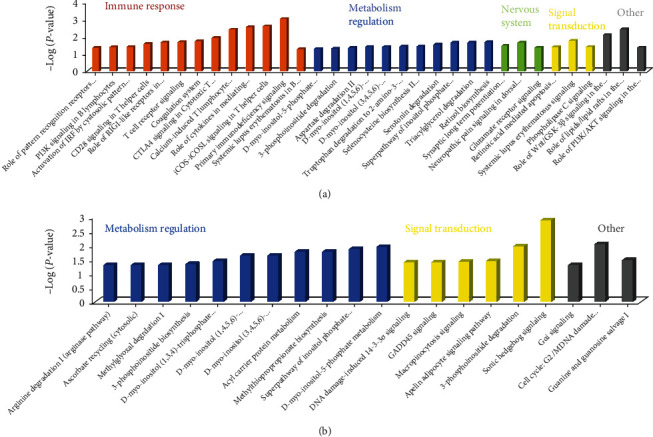
Ingenuity Pathway Analysis (IPA) annotates the canonical pathway analysis of differentially expressed mRNAs. These differentially expressed mRNAs were regulated by differentially expressed lncRNAs between PDC and BC (Pearson's correlation coefficient ∣PCC | >0.7 and *P* < 0.05) ((a) 115 upregulated mRNAs; (b) 321 downregulated mRNAs).

The differentially expressed mRNA-lncRNA formed coexpression network (CNC) (∣PCC | >0.7 and *P* < 0.05) consists of 421 DEmRNAs and 357 DElncRNAs (Table S2). These were 421 DEmRNAs (102 upregulated and 319 downregulated) in CNC subjected to the Ingenuity Pathway Analysis (IPA). The 102 upregulated mRNAs generated 33 significantly enriched pathways (*P* < 0.05) (Figure 2(a)), which were involved in metabolic regulation and immunizing inflammatory reaction, such as the T cell receptor signaling (*P* = 0.01), retinol biosynthesis (*P* = 0.02), and triacylglycerol degradation (*P* = 0.02). Meanwhile, we identified 20 IPA pathways (*P* < 0.05) from 319 downregulated mRNAs (Figure 2(b)), most of which were associated with the metabolism of lipids/lipoproteins (such as 3-phosphoinositide degradation, acyl carrier protein metabolism, and D-myo-inositol-5-phosphate metabolism). We subsequently classified these 20 pathways into functional groups that were the metabolism (12/20), signal transduction (5/20) pathways, and so on. These data indicated that individuals with PDC showed specific gene expression profiles and lncRNA expression features in peripheral blood. Among them, ARHGAP9 has been found to lead to endothelial dysfunction in patients with coronary spastic angina [38]. These results suggest that the majority of pathways with DElncRNAs were consistent with these pathways of DEmRNAs.

**Figure 3:**
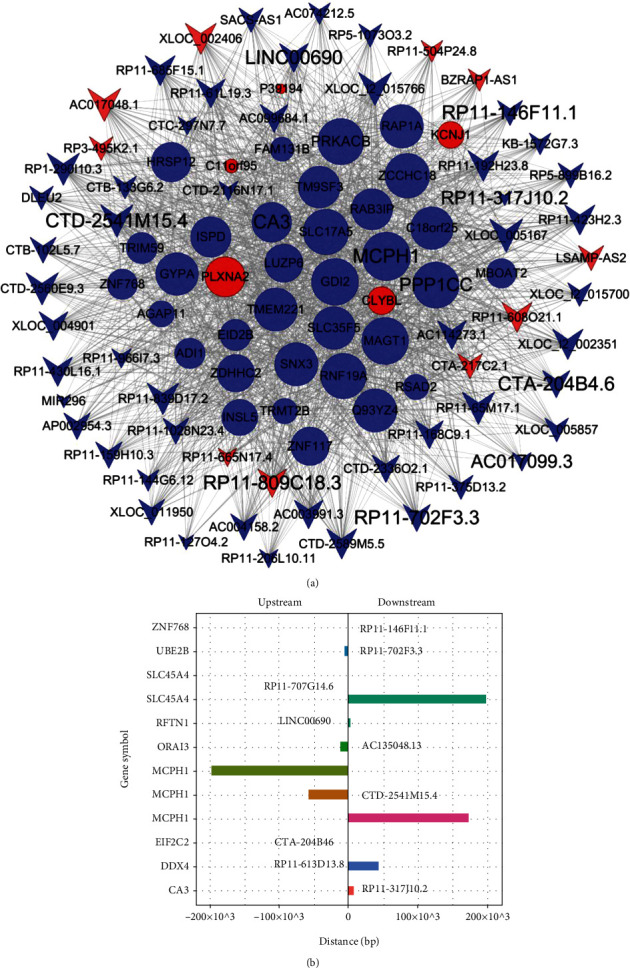
Target gene prediction of differentially expressed lncRNAs between PDC and BC. (a) The differentially expressed mRNAs and lncRNAs from Figure 2 were constructed into the coding-noncoding gene network (degree > 60). Red represents upregulated and blue represents downregulated genes. Size represents the importance of a node (degree). The edge denotes the interaction strength. Circles and inverted triangles represent genes and lncRNAs, respectively. (b) Distances between lncRNAs and their predicted target genes (<300 kb). The left vertical axis shows the gene symbol of the coding genes, on the right side of each row, that is, the corresponding gene symbol of lncRNA. The pathways of these mRNAs might mainly be related to lipid metabolism.

Next, we explored which key lncRNAs regulated the mRNA of the above-mentioned. According to the correlation coefficient analysis in Table S2, in order to further focus on these lncRNAs and their target genes, lncRNA-mRNA coexpression network showed that 58 lncRNAs (10 upregulated and 48 downregulated lncRNAs), which were at the central positions of the network (degree centrality > 60), might play key roles in regulating metabolism pathways (Figure 3(a)). There are 38 DEmRNAs (5 upregulated and 33 downregulated), which were regulated by these 58 lncRNAs, which were mostly related to metabolic pathways (such as lipid metabolism and fatty acid metabolism). Especially, nearly half of the pathways (12/35) were related to metabolic regulation in the downregulated genes, which was consistent with the proportion of lipid metabolic pathways in Figure 2(b) (12/20). Among them, GYPA is the high-degree gene (it is regulated by multiple lncRNAs) that affects the expression of glycophorin A, and it is an important indicator of carotid atherosclerotic lesions [39].

If the genome position of the mRNA-encoding genes is adjacent to that of the coexpressed lncRNA-encoding gene, it suggests that the lncRNA regulates the expression of the mRNA. By combining the lncRNA-mRNA coexpression network and the targets of lncRNA in upstream or downstream 300 kb of lipid metabolism and immune inflammation-related pathways, we identified a total of 12 lncRNA transcripts and their predicted regulatory protein-coding genes (<300 kb and ∣PCC | >0.7) (Figure 3(b)). Among these 12 lncRNA-mRNA pairs, these data provided valuable clues about these lncRNAs and their nearby coding genes in metabolic disorders of subjects with PDC (Figures 3(a) and 3(b)).

### 3.3. The Target Gene Prediction of lncRNAs in the Lipid Metabolism Pathway of PDC

**Figure 4:**
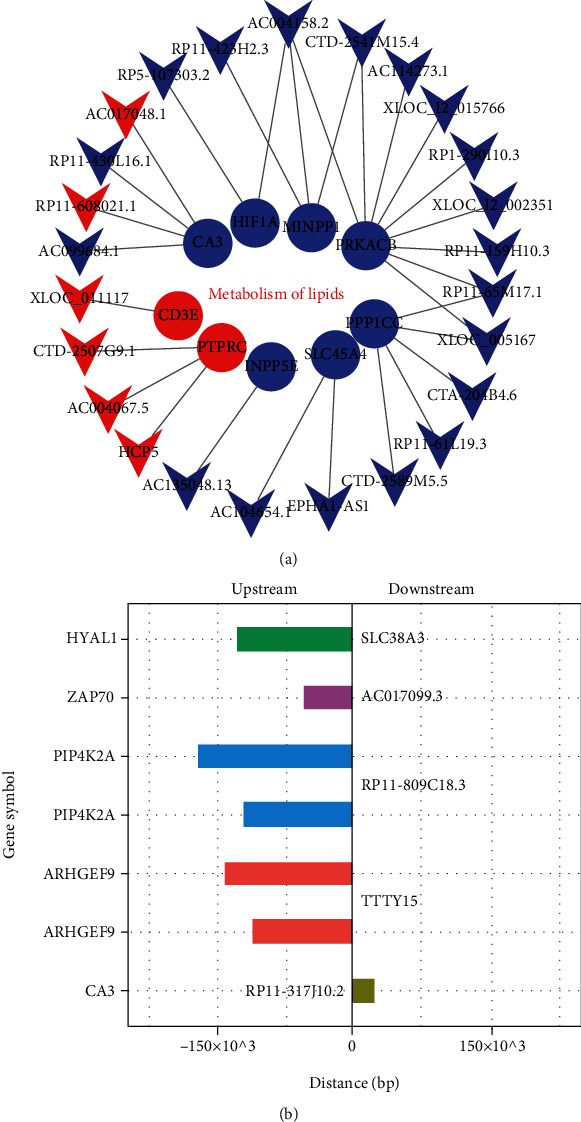
Target gene prediction of lncRNAs in the lipid metabolism pathway. (a) Coexpression network of the differentially expressed lncRNAs and genes in the lipid metabolism pathway. Red represents upregulated and blue represents downregulated genes. Circles and inverted triangles represent genes and lncRNAs, respectively. (b) Regulation of differentially expressed lncRNAs to nearby coding genes in the lipid metabolism pathway (<300 kb). The left vertical axis shows the gene symbol of the coding genes, on the right side of each row, that is, the corresponding gene symbol of lncRNA.

Figure 3 reveals that lipid metabolism accounted for a large proportion of these pathways. To find out which lncRNAs regulated these genes in the lipid metabolism pathway, we constructed a coexpression network for these mRNAs and corresponding lncRNAs in subjects with PDC. Next, we selected the top 30 pairs of lncRNA-mRNA with the lowest *P* to map the coexpression network, including nine mRNAs and 25 lncRNAs (Figure 4(a)). The functions of these mRNAs were mostly related to pathways of metabolism of lipids and/or lipoproteins. It indicated that these nine mRNAs (two upregulated and seven downregulated mRNAs) in the lipid metabolism pathway were mainly regulated by these DElncRNAs (25 genes). However, little is known about the functions of lncRNAs and lncRNA-mRNA interactions, which should be further studied in the future.

Then, we further analyzed how these dysregulated lncRNAs regulate mRNAs in the lipid metabolism pathway [22]. We chose 26 DEmRNAs to hunt their nearby DElncRNAs in the lipid metabolism pathway in the PDC. The coexpressed protein-coding genes were defined as target genes with one DElncRNA within 300 kb upstream and downstream on the same chromosome. The filtering results are shown in Figure 4(b) (including seven lncRNA transcripts and their predicted regulatory protein-coding genes). Each lncRNA had a different number of neighboring coding genes. For example, RP11-317J10.2 had only one nearby coding gene. In contrast, lncRNAs TTTY15 and RP11-809C18.3, respectively, had two nearby coding genes (Figure 4(b)). It indicated that these neighboring genes might be regulated by lncRNAs.

### 3.4. Validation of Deregulated lncRNAs and mRNAs by RT-qPCR in Independent 21 Subjects of PDC and BC

**Figure 5:**
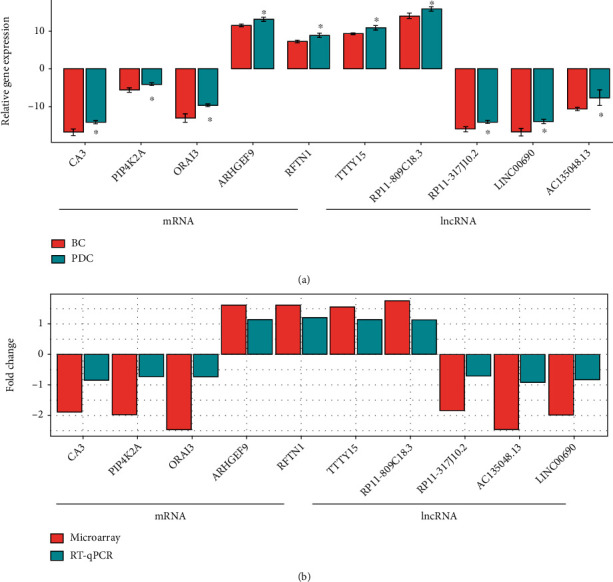
Validation for the expression of significant transcripts by quantitative RT-qPCR. (a) RT-qPCR verification of the expression profiles of differentially expressed lncRNAs/mRNAs. PDC and BC represent the phlegm-dampness constitution and balanced constitution, respectively. (b) Comparison of RT-qPCR and microarray analysis data. The *x*-axis and *y*-axis present mRNA/lncRNA name and relative expression (fold change), respectively. Fold change > 0 and <0 represents the upregulation and downregulation, respectively. ^∗^*P* < 0.05; ^∗∗^*P* < 0.01.

**Figure 6:**
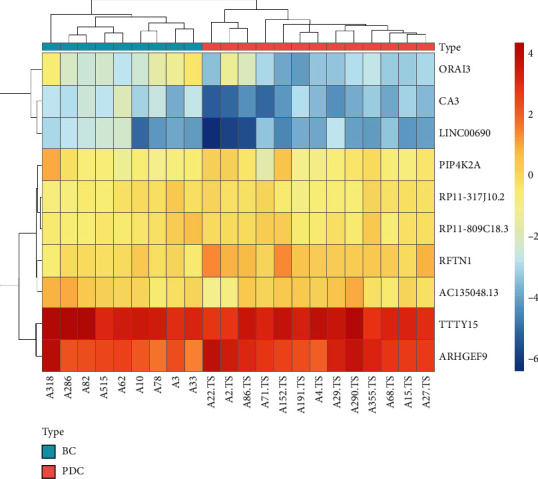
The heatmap of 5 lncRNA-mRNA pairs is of the expression of significant transcripts by quantitative RT-qPCR. Row and column represent differentially expressed lncRNAs/mRNAs and samples, respectively, in the heatmaps (*P* < 0.05, ∣fold change | ≥1.5) in phlegm-dampness constitution (PDC) compared with Balanced constitution (BC). Red and blue indicate up- and downregulation, respectively. -6, -2, 0, 2, and 4 represent fold changes in the corresponding spectrum.

To verify the reliability of the microarray data, some key functional genes of metabolic disorders, including five DElncRNAs and five DEmRNAs from Figures 3(b) and 4(b), were confirmed in 21 subjects with PDC and BC by RT-qPCR, as shown in Figure 5. The relative expressions of CA3, ORAI3, PIP4K2A, RP11-317J10.2, LINC00690, and AC135048.13 were significantly downregulated (*P* < 0.05) in the blood of the subjects with PDC, compared to those with BC. In contrast, the relative expressions of RFTN1, ARHGEF9, TTTY15, and RP11-809C18.3 were upregulated in the blood of the subjects with PDC. Therefore, the RT-qPCR data verified the veracity of microarray results (Figures 5(a) and 5(b)). The finding provided reliable evidence that these lncRNAs and mRNAs could be implicated in the pathogenesis of metabolic diseases in PDC. A biomarker means a panel that could distinct case and control with as few features as possible. These 5 lncRNA-mRNA pairs could be used as potential biomarkers that they distinct BC and PDC. The heatmap of 5 lncRNA-mRNA pairs is of the expression of significant transcripts by quantitative RT-qPCR (Figure 6).

## 4. Discussion

In the present study, we identified several interaction pairs of lncRNA-nearby targeted mRNAs, such as RP11-317J10.2-CA3, RP11-809C18.3-PIP4K2A, LINC0069-RFTN1, TTTY15-ARHGEF9, and AC135048.13-ORAI3. Meanwhile, individuals with PDC exhibited transcriptional characteristics regarding lipid metabolism and immune inflammation-related pathways. These findings were consistent with the observations of profuse phlegm, fat body, loose abdomen, and greasy face in individuals with PDC. In short, our study first identified several differentially expressed lncRNAs and mRNAs and suggested that their interactions played a crucial role in the process of metabolic dysregulation in the PDC.

RP11-317J10.2 is identified in human blood as an antisense lncRNA [40]. In our study, we found that RP11-317J10.2 was the most downregulated lncRNA in the blood of the subjects with PDC. Furthermore, downregulated CA3 was the nearby targeted mRNA of RP11-317J10.2. It is well known that CA3 is one of the diagnostic markers of perioperative MI during coronary bypass surgery [41]. Our results indicated that the downregulation of CA3 and RP11-317J10.2 might be involved in lipid metabolism, which could provide a new therapeutic regimen of metabolic diseases in the PDC.

It is found that RP11-809C18.3 is an antisense lncRNA in the PDC [42]. In the present study, we first found the upregulation of RP11-809C18.3 in the blood of the subjects with PDC. Besides, upregulated PIP4K2A was the nearby targeted mRNA of RP11-809C18.3. It has been observed that the expression of PIP4K2A is significantly increased in subjects with PDC, which regulates intracellular cholesterol transport through modulating PI(4,5)P2 homeostasis [43]. Additionally, noncoding RNA RP11-809C18.3 regulates PIP4K2A, which is related to phospholipid metabolism by modulating PI(4,5)P2 homeostasis [44, 45]. It has been proposed that increased expressions of RP11-809C18.3 and PIP4K2A may promote inositol phosphate metabolism, which contributes to the process of PDC.

Up to now, only a few articles have investigated LINC00690 in the PDC. Herein, we found that LINC00690 was downregulated in the blood of the subjects with PDC. Furthermore, upregulated RFTN1 was the nearby targeted mRNA of LINC00690. It is pointed out that RFTN1 is involved in controlling lipopolysaccharide-induced TLR4 internalization and TICAM-1 signaling in a cell type-specific manner [46]. A relevant study of RFTN1 has revealed that common body mass index-associated variants confer the risk of extreme obesity [47]. Our findings indicated that the downregulation of LINC00690 and the upregulation of RFTN1 played a key role in the metabolic disorders of PDC. However, further research is required to validate the deeper function of LINC00690 and RFTN1 in the PDC.

Among the dysregulated mRNAs, ARHGAP9 was the nearby targeted mRNA of TTTY15. It has been demonstrated that TTTY15 plays an important role in regulating hypoxia-induced vascular endothelial cell injury [48]. Knockdown of long noncoding RNA TTTY15 helps protect cardiomyocytes in case of hypoxia-induced apoptosis and mitochondrial energy metabolism dysfunction via TTTY15/let-7i-5p and TLR3/NF-*κ*B pathways [49]; and TTTY15 knockdown also protects cardiomyocytes from hypoxia-induced injury by regulating the let-7b/MAPK6 axis [50] or by regulating the miR-98-5p/CRP pathway [51]. Moreover, the polymorphism of ARHGAP9 is associated with coronary artery spasm [38]. Herein, we found the interaction between TTTY15 and ARHGAP9 in the PDC. Our result suggested that upregulated TTTY15 and ARHGAP9 may play a vital role in metabolic disease, which could be used as PDC biomarkers to assist in clinical diagnosis.

AC135048.13 is associated with the occurrence and recurrence of myocardial infarction [52]. In this study, we first found the top 10 downregulated expressions of AC135048.13 in the blood of the subjects with PDC. Besides, downregulated ORAI3 is associated with the changes in cerebrovascular contractile responses in high-salt intake-induced hypertension. The expression of ORAI3 proteins has been linked to vascular and airway pathologies, including restenosis, hypertension, and atopic asthma [53]. ORAI3 was the nearby targeted mRNA of AC135048.13. It has been demonstrated that ORAI3 plays an important role in patients with cardiac hypertrophy [54]. Herein, the interaction between AC135048.13 and ORAI3 suggested that decreased expression of AC135048.13 and ORAI3 was involved in the lipid metabolism of the PDC. These findings provided solid evidence that these lncRNAs and mRNAs could be implicated in the pathogenesis of metabolic disorders with the PDC. Furthermore, we speculated that these lncRNAs played a potential regulatory role in the metabolic regulation of phlegm-dampness-susceptible diseases.

Previous studies have found that subjects with PDC have specific mRNA expression signatures and methylation profiles [12, 14]. As indicated in Fig. S2, the reproducibility of the pathway was studied between this study and a previous study by IPA. It showed that metabolism-related pathways accounted for a large proportion of the two studies. Consistent with the clinical observation that subjects with PDC tend to develop a metabolic disorder, series-clustering analysis detected several key lipid metabolic genes (PRKAR1A, ACSL4, SMPD3, etc.) [10, 12]. This study also confirmed that subjects with PDC were a population with a dysregulation tendency of metabolic disorder-related mRNAs (Figure 2).

Nowadays, most studies have focused on the pathological study of metabolic disease, while only a few have been carried out on the prevention of metabolic diseases [55, 56]. Previously, Ma and coworkers have reported that lncRNAs may regulate diverse gene expressions, which are roughly summarized to epigenetic, transcriptional, and posttranscriptional levels in cardiovascular diseases [22]. One of the principles for “P4 medicine” is the preventive treatment of disease. The “P4 medicine” plays an important role in the diagnosis, prevention, and treatment of metabolic disease [5]. This study explains which people are more likely to have metabolic diseases, indicating that the research of the PDC is a good pointcut to explore the undiseased state of metabolic disorders. When PDC develops to metabolic diseases, it will still have the characteristics of PDC. The PDC is a susceptibility factor for metabolic diseases and coexists with metabolic diseases. According to the results of epidemiological studies, the occurrence of metabolic diseases is related to the phlegm-dampness constitution (PDC) (*P* < 0.001) [57], which is positively associated with the level of PDC (*P* < 0.001) [9]. Compared with the balanced constitution (BC), the patients of PDC are more likely to have hypertension and hyperlipidemia. Moreover, the use of peripheral blood is a suitable method to identify surrogate markers in TCM constitutions. Liew et al. have compared the peripheral blood transcriptome with genes expressed in nine different human tissue types [58]. They have found that expression of over 80% is shared in any given tissue, and tissue-specific gene transcripts can be detected in circulating blood cells. Another advantage of peripheral blood is that it can be dynamically monitored in real time, which is very convenient. Furthermore, there are several limitations in the present study. First, we need further experiments to gain mechanistic insights. Related research should be viewed in the future. Second, a larger sample size is necessary for further in vivo and in vitro validation of identified differentially lncRNAs and mRNAs. Third, lncRNAs and mRNAs in exosomes might be a better choice in the future.

## 5. Conclusions

Our study identified five pairs of lncRNA and mRNA that could be used as potential biomarkers for the PDC, including RP11-317J10.2-CA3, RP11-809C18.3-PIP4K2A, LINC0069-RFTN1, TTTY15-ARHGEF9, and AC135048.13-ORAI3. And lipid metabolic disorders could be associated with the PDC. In conclusion, these results indicated several dysregulated lncRNAs that were potentially associated with the development and progression of metabolic diseases in the PDC. These findings provided valuable insights into novel therapeutic strategies for the prevention and treatment of metabolic disorders.

## Figures and Tables

**Table 1:** Diagnostic standards for the phlegm-dampness constitution (PDC) and balanced constitutions (BC).

Variables	PDC	BC
Main characteristics	Profuse phlegm	Energetic
Secondary characteristics	Fat body	Without any symptoms or characteristics of other constitutions
	Loose abdomen	Eat well
	Greasy face	Sleep well
	Chest distress	Good posture
	Sticky and sweet taste in the mouth Slippery pulse	The tongue is pale red with thin white coating
	The tongue is fat and white with coating	

**Table 2:** Demographic and clinical characteristics of subjects.

Variables	PDC (*n* = 13)	BC (*n* = 9)	*P*-value
Male, *n* (%)	46.15	44.44	0.94
Age (years), mean (SD)	37 ± 6.78	37 ± 7.38	0.78
Body mass index (kg/m^2^)	23.27 ± 1.26	22.86 ± 3.05	0.12

## Data Availability

The datasets used and/or analyzed during the current study would be available from the first author and corresponding author on reasonable request. The raw data of this study were uploaded to the NCBI GEO database (NCBI GEO accession number GSE158042).

## Supplementary Materials

Supplementary Materials Fig. S1: box plots summarizing the lncRNA and mRNA profiles. After normalization, the distributions of the log2 ratios of fluorescence signals in phlegm-dampness constitution (PDC) samples were nearly identical among balanced constitution (BC) samples. Fig. S2: the reproducibility of the pathway was researched by IPA between this study and previous studies. The li_up and li_down, respectively, represent the upregulated and downregulated mRNAs of Wang's paper [1]. Table S1: lncRNA RT-qPCR primers and product sizes. Table S2: the top 100 coexpression networks (CNC) of 421 differentially expressed mRNAs and 357 lncRNAs were involved (*P* < 0.05, ∣PCC | >0.7). PCC: Pearson's correlation coefficient. Table S3: demographic and clinical characteristics of 21 subjects.
