# Supplementary material for: Analyses of Long Noncoding RNA and mRNA Profiles in Subjects with the Phlegm-Dampness Constitution
Source: Biomed Res Int. 2021 Dec 10;2021:4896282. doi: 10.1155/2021/4896282 (PMC8683173; doi:10.1155/2021/4896282)
Supplement: Supplementary Materials — Fig. S1: box plots summarizing the lncRNA and mRNA profiles. After normalization, the distributions of the log2 ratios of fluorescence signals in phlegm-dampness constitution (PDC) samples were nearly identical among balanced constitution (BC) samples. Fig. S2: the reproducibility of the pathway was researched by IPA between this study and previous studies. The li_up and li_down, respectively, represent the upregulated and downregulated mRNAs of Wang's paper [1]. Table S1: lncRNA RT-qPCR primers and product sizes. Table S2: the top 100 coexpression networks (CNC) of 421 differentially expressed mRNAs and 357 lncRNAs were involved (P < 0.05, ∣PCC | >0.7). PCC: Pearson's correlation coefficient. Table S3: demographic and clinical characteristics of 21 subjects. [file 4896282.f1.pdf]

Supplementary material

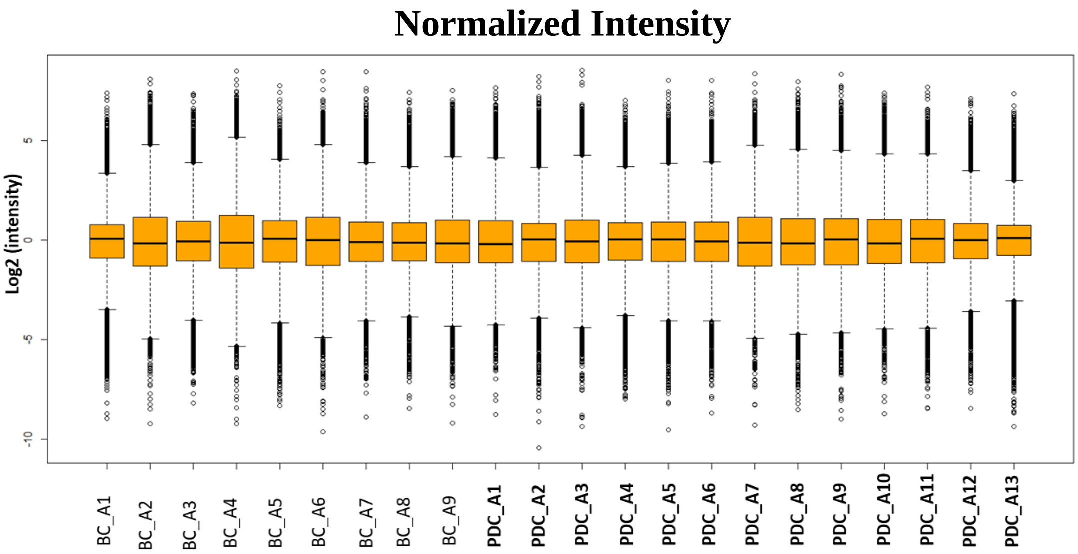

Fig. S1. Box plots summarizing the lncRNA and mRNA profiles. After normalization, the distributions of the log2-ratios of fluorescence signals in phlegm-dampness constitution (PDC) samples were nearly identical among balanced constitution (BC) samples.

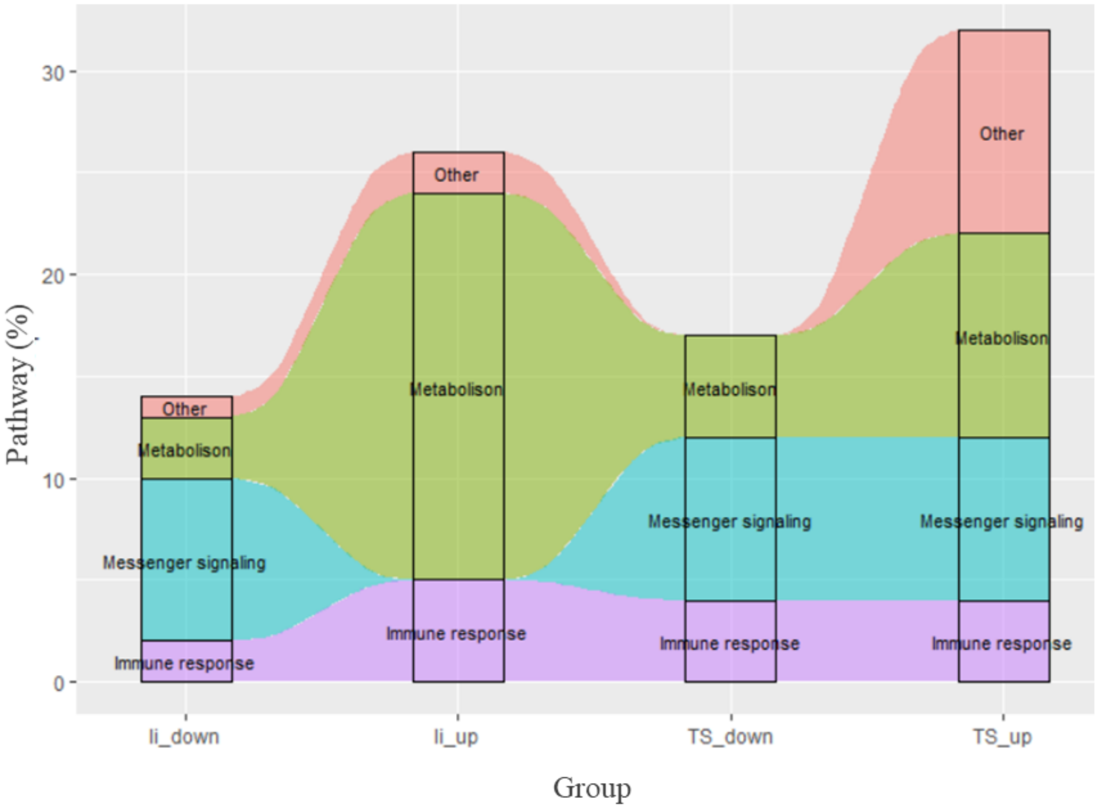

Fig. S2. The reproducibility of the pathway was researched by IPA between this study and previous studies. The TS_up and TS_down respectively represents the up-regulated and down-regulated mRNAs of this study. The li_up and li_down respectively represents the up-regulated and down-regulated mRNAs of L. Li's paper [1].

References

[1] L. Li, J. Feng, H. Yao, L. Xie, Y. Chen, L. Yang, S. Hou, S. Zhao, R. Sun, Y. Wu, T. Bai, Y. Li, R. Yu, J. Wang, Q. Wang, Gene expression signatures for phlegm-dampness constitution of Chinese medicine, Sci China Life Sci 60(1) (2017) 105-107.

Table S1. LncRNA RT- qPCR primers and product sizes.

lncRNA ID	Forward primer (5′- >3′)	Reverse primer (5′- >3′)	Size of PCR Product (bp)
CA3	TCTCCAGTGCTGAGAACGA	CTCACCACCCTGTTATTGAT	82
RFTN1	CTCCTTAGACCACCCGAC	ACTGAGGTATAACACCAACGA	86
PIP4K2A	ACCGGCTTAATGTTGATGG	CTGTAGAGCCCTTTAAGTCGTA	101
ORAI3	CTTTGCCCTGCATTTCTACC	AGGCGATTCAGTTCCTCT	81
ARHGEF9	TATGATGATGCTGGAGTGGAC	AGAAGTGCTGCAACATGG	93
TTTY15	GTATGATTGGCCTTAGAGCAC	TCATCCTATCAACTGACCTTCC	85
RP11-317J10.2	ACTGTGCTTACAGGGCCTA	CCGTGGATGGAGTCAAGT	88
LINC00690	AGATCTTCCAAGTCCAGCCA	AGAGGGAAGGGATGTGTC	94
RP11-809C18.3	TGGGGAATTCTGGCTGAG	TCAGCACAAATGTTAGCATTGA	118
AC135048.13	TAGCCACCATTTCCATTCCTAT	CTGGGAAACAGACTGTCTCA	97

Table S2. **The top 100 co-expression network (CNC) of 421 differentially expressed mRNAs and 357 lncRNAs were involved (P < 0.05, |PCC| > 0.7).** (PCC: Pearson correlation coefficient).

mRNA	LncRNA	Correlation	P-value
SLC45A4	RP11-707G14.6	0.9895119	1.36E-10
CARD17	LINC00703	0.984012	1.36E-09
GDI2	AC004158.2	0.9838924	1.42E-09
TRH	CTB-102L5.7	0.9826928	2.10E-09
MCPH1	CTD-2541M15.4	0.9826714	2.12E-09
TRH	SACS-AS1	0.9805585	3.97E-09
HRSP12	RP11-61L19.3	0.9782015	7.42E-09
C17orf78	RP11-331F9.3	0.976173	1.21E-08
Q93YZ4	RP11-168C9.1	0.9745898	1.71E-08
FAM90A1	RP11-482D24.3	0.9731323	2.32E-08
TTC39C	AC004067.5	0.97306	2.35E-08
CARD8	MIR296	0.9711006	3.45E-08
CEP350	RP11-144G6.12	0.9710308	3.50E-08
TTC39C	CTD-2507G9.1	0.9684739	5.54E-08
LENG8	CTB-102L5.7	0.968213	5.79E-08
KRTAP13-3	RP11-718I15.1	0.9674175	6.63E-08
LACTB2	AC004158.2	0.965052	9.70E-08
MCPH1	CTD-2336O2.1	0.9649822	9.81E-08
KRTAP13-3	AC023128.1	0.9643445	1.08E-07
PEX13	RP11-820L6.1	0.9630746	1.31E-07
POLE2	RP11-144G6.12	0.9627661	1.37E-07
LENG8	RP11-168C9.1	0.9611557	1.72E-07
LACTB2	DLEU2	0.9607576	1.82E-07
PRKACB	RP11-159H10.3	0.9603007	1.94E-07
MCPH1	RP11-65M17.1	0.9601132	1.99E-07
FLJ44838	RP11-864I4.3	-0.960045	2.01E-07

RAB3IP	RP11-192H23.8	0.9596123	2.13E-07
LUZP6	CTD-2589M5.5	0.9593015	2.22E-07
SNX3	CTD-2589M5.5	0.9590293	2.30E-07
KRTAP13-3	CTD-2532D12.4	0.9583467	2.51E-07
ORAI3	AC135048.13	0.9580502	2.61E-07
ZCCHC18	AC004158.2	0.9571166	2.94E-07
OAT	RP11-1028N23.4	0.9558822	3.43E-07
RNF19A	CTD-2589M5.5	0.9558968	3.43E-07
PER3	CTC-304I17.5	0.955858	3.44E-07
GDI2	ZCCHC18	0.9553168	3.68E-07
ATF6B	KB-1572G7.3	0.9549737	3.83E-07
TMEM221	CTD-2541M15.4	0.9546193	4.00E-07
KRTAP13-3	RP11-167P22.3	0.9544495	4.08E-07
Q93YZ4	CTB-102L5.7	0.9520167	5.41E-07
PEX13	AC023128.1	0.9508375	6.17E-07
LENG8	KB-1572G7.3	0.9508131	6.18E-07
ZNF117	RP11-159H10.3	0.9505038	6.40E-07
PRKACB	CTD-2541M15.4	0.9504253	6.45E-07
TRH	RP11-168C9.1	0.9501974	6.61E-07
NCAPG2	CTB-133G6.2	0.9476456	8.66E-07
PPP1CC	CTD-2589M5.5	0.9471303	9.13E-07
DAZAP1	ZCCHC18	0.9468264	9.42E-07
SLC17A5	AC004158.2	0.9461139	1.01E-06
MCPH1	RP1-290I10.3	0.9457305	1.05E-06
C18orf25	CTD-2541M15.4	0.9456015	1.06E-06
ZDHHC2	CTA-204B4.6	0.9455959	1.07E-06
GJD2	AC099344.3	0.9448587	1.15E-06
HIP1R	RP11-383C6.2	0.9447342	1.16E-06
PRKACB	AC114273.1	0.9440662	1.24E-06

GDI2	CTD-2541M15.4	0.9437577	1.27E-06
DTX3	RP11-705O3.1	0.9429145	1.38E-06
YOD1	RP11-702F3.3	0.9424639	1.44E-06
CLIC2	RP5-1073O3.2	0.941823	1.53E-06
RAB3IP	AC099684.1	0.9417726	1.54E-06
HIP1R	XLOC_001441	0.9415231	1.57E-06
HRSP12	RP11-430L16.1	0.9412279	1.62E-06
FBXO5	RP11-375D13.2	0.9409165	1.66E-06
GYPA	AC017048.1	-0.940656	1.70E-06
C8orf80	RP11-215N21.1	-0.940543	1.72E-06
LACTB2	RP11-144G6.12	0.9403463	1.75E-06
SLC17A5	ZCCHC18	0.9400605	1.80E-06
Q93YZ4	RP11-685F15.1	0.9395887	1.87E-06
LRRIQ1	CTD-2532D12.4	0.9394809	1.89E-06
PRKACB	AC004158.2	0.9395072	1.89E-06
PRKACB	RP1-290I10.3	0.9391295	1.95E-06
SNX3	RP11-702F3.3	0.939058	1.96E-06
TDO2	CTA-217C2.1	0.9388402	2.00E-06
ZDHHC2	RP11-61L19.3	0.9388561	2.00E-06
GDI2	DLEU2	0.9387404	2.02E-06
ZNF304	RP11-375D13.2	0.9387363	2.02E-06
C17orf78	CT64	0.9379785	2.16E-06
ARL6IP1	CTD-2589M5.5	0.937815	2.19E-06
Q93YZ4	RP11-839D17.2	0.9376711	2.22E-06
MFHAS1	CTD-2589M5.5	0.9374868	2.25E-06
CAMTA1	RP11-1028N23.4	0.9369385	2.36E-06
FAM185A	RP11-144G6.12	0.9366641	2.42E-06
SLC5A7	RP11-718I15.1	0.9364262	2.47E-06
HIP1R	RP11-125B21.2	0.9362374	2.50E-06

C18orf25	RP11-423H2.3	0.9359343	2.57E-06
C17orf78	LINC00264	0.9357893	2.60E-06
HPRT1	XLOC_l2_012378	0.9354959	2.67E-06
ZCCHC18	XLOC_005857	0.934913	2.80E-06
PRKACB	RP11-65M17.1	0.9348794	2.81E-06
FBXO30	CTD-2589M5.5	0.9347795	2.83E-06
DAZAP1	AC004158.2	0.934507	2.89E-06
MCPH1	AC004158.2	0.9345314	2.89E-06
INSL5	CTB-133G6.2	0.9338976	3.04E-06
MINPP1	AC004158.2	0.9335128	3.14E-06
FBXO30	LINC00849	0.9330637	3.25E-06
MCPH1	RP11-159H10.3	0.9327146	3.34E-06
POLE2	DLEU2	0.9325059	3.40E-06
FAM200B	RP11-1028N23.4	0.9324354	3.42E-06
ACSL3	RP11-1028N23.4	0.9323484	3.44E-06
ZCCHC18	RP5-899B16.2	0.9317486	3.61E-06

Table S3. **Demographic and clinical characteristics of 21 subjects.**

Variables	PDC (n = 9)	BC (n = 12)	P-value
Male, n (%)	42.66	58.33	0.56
Age (years), Mean (SD)	36 ± 6.78	38 ± 5.68	0.36
Body mass index (kg/m^2)	23.12 ± 1.9	22.27 ± 2.3	0.25
